# Spectral Reflectance Estimation from Camera Responses Using Local Optimal Dataset

**DOI:** 10.3390/jimaging9020047

**Published:** 2023-02-17

**Authors:** Shoji Tominaga, Hideaki Sakai

**Affiliations:** 1 Department of Computer Science, Norwegian University of Science and Technology, 2815 Gjøvik, Norway; 2 Department of Business and Informatics, Nagano University, Ueda 386-0032, Japan; 3 Professor Emeritus, Kyoto University, Kyoto 606-8501, Japan

**Keywords:** surface-spectral reflectance, reflectance estimation, multispectral imaging, local optimal dataset, statistical estimation method, mathematical programming method

## Abstract

A novel method is proposed to estimate surface-spectral reflectance from camera responses using a local optimal reflectance dataset. We adopt a multispectral imaging system that involves an RGB camera capturing multiple images under multiple light sources. A spectral reflectance database is utilized to locally determine the candidates to optimally estimate the spectral reflectance. The proposed estimation method comprises two stages: (1) selecting the local optimal reflectance dataset and (2) determining the best estimate using only the local optimal dataset. In (1), the camera responses are predicted for the respective reflectances in the database, and then the prediction errors are calculated to select the local optimal dataset. In (2), multiple methods are used; in particular, the Wiener and linear minimum mean square error estimators are used to calculate all statistics, based only on the local optimal dataset, and linear and quadratic programming methods are used to solve optimization problems with constraints. Experimental results using different mobile phone cameras show that the estimation accuracy has improved drastically. A much smaller local optimal dataset among spectral reflectance databases is enough to obtain the optimal estimates. The method has potential applications including fields of color science, image science and technology, computer vision, and graphics.

## 1. Introduction

Surface-spectral reflectance is typically defined as the ratio of light reflected from an object’s surface to the incident light as a function of wavelength. It provides physical features that are inherent and discriminative to the surfaces of objects composed of different materials, such as natural and man-made objects. Hence, knowledge regarding the spectral reflectance of objects can be applied extensively to color science, image science and technology, computer vision, and computer graphics. Problems in estimating the surface-spectral reflectance from camera responses have been investigated concurrently with the development of cameras and imaging systems; consequently, many methods have been proposed.

Spectral reflectance estimation methods based on camera responses can be classified into two primary approaches: model-based approach [1,2,3,4,5,6,7,8,9,10,11,12,13] and training (or learning)-based approach [14,15,16,17,18,19,20,21,22,23,24,25,26].

In the model-based approach, camera responses are described using camera spectral sensitivities, surface-spectral reflectance, and illuminant spectral power distributions. This approach is the more typically used approach and includes finite-dimensional modeling methods [1,3] and Wiener estimation methods [4,5,6,7,8,9,10,11,12]. The Wiener estimation methods are based on a statistical approach, in which noise in the imaging system and a certain statistic of the spectral reflectance are considered. The Wiener estimation methods are one of the most typically used and reliable methods when the spectral sensitivity functions of the imaging system are known. Recently, an improved estimation method, known as the “linear minimum mean square error (LMMSE)” method [13] was proposed to minimize the estimation error of spectral reflectance. Its estimation accuracy was theoretically verified to be better than that of the conventional Wiener method.

The training-based approach is typically constructed without knowledge regarding the camera spectral sensitivities and illuminant spectral distributions; instead, a large training dataset, which is a large table comprising a pair of camera responses and the corresponding spectral reflectances, is used. Regression methods directly establish the relationship between RGB responses and spectral reflectances and include support vector regression [17,18], kernel regression [19,20], linear regression [21,22], and neural networks [26]. This method does not necessitate the assumption of a linear relationship between RGB values and the spectral sensitivity function and allows the camera response to be mapped nonlinearly to the spectral sensitivity function. Therefore, raw camera data are not required. However, this approach requires a significant amount of training data to establish reliable mapping and can be utilized solely for a specific imaging system. The camera responses are acquired under specific conditions of the illumination environment and imaging geometries, and the corresponding spectral reflectances are measured using a spectrometer or obtained using a spectral imaging device. Thus, the training data depend on the illumination environment, unless an additional operation such as white balance is implemented [24,25].

Recently, software that can store images captured as raw data has become publicly available when using digital single-lens reflex (DSLR) cameras and mobile phone cameras. The spectral sensitivity function represents the image sensor output per unit of incident light energy at each wavelength within the spectral range in which the camera system operates. This function results in a linear relationship between the camera inputs and outputs using raw image data [27]. Under these conditions, spectral sensitivity can be measured using monochromatic light. The measured spectral sensitivities for different cameras have been published [27,28]; for instance, databases for 28 DSLR cameras [29] and 20 mobile phone cameras [30] are currently available. Considering recent imaging environments on software development and database availability, as the model-based approach does not require prior training, it can be a more direct estimation method for estimating surface-spectral reflectances.

In this paper, we propose a novel method to effectively use a reflectance database and significantly improve the estimation accuracy of spectral reflectance from image data acquired using an RGB digital camera. The imaging system is a multispectral image acquisition system extended from a simple RGB system, where an RGB camera captures multiple images of an object scene under multiple light sources with different illuminant spectra in the visible range. We adopt a general imaging model, in which the camera responses are described by combining the camera spectral sensitivities, surface-spectral reflectance, illuminant spectral power distributions, additive noise terms, and gain parameters.

We do not use the entire data of the spectral reflectance database, but effectively select a subset of the database to optimally estimate the spectral reflectance. Similar ideas were proposed for recovering spectral reflectance from tristimulus values [31] and for adaptively reconstructing spectral reflectance based on the Wiener estimator from image data [7,11]. In this study, we estimate the spectral reflectance from image data in two stages. In the first stage, a set of the most reliable candidates for the optimal estimation of the spectral reflectance is selected from the reflectance database based on observations of a target object surface, which is known as the *local optimal reflectance dataset*. In the second stage, the best reflectance is estimated using only the local optimal dataset. We present multiple estimation algorithms that can provide the best estimates, i.e., those that minimize the estimation error. One class of algorithms comprises the statistical method, where the mean values, autocorrelation matrices, and covariance matrices are calculated from locally extracted optimal data only and then used as the statistical characteristics to describe the best estimate. Another class comprises a mathematical programming method that uses local optimal reflectances and solves the optimization problem to minimize the prediction error under weight constraints.

In the following, Section 2 describes an observation model for a multispectral image acquisition system that uses an RGB camera and multiple light sources. The model is constructed with three spectral functions, i.e., known camera spectral sensitivities, known illuminant spectra, and unknown surface-spectral reflectance, and includes two parameters, i.e., gain and independent noise.

Section 3 describes the selection of a local optimal reflectance dataset. The camera responses recorded from an actual object are compared with observations predicted from the respective spectral reflectance in the reflectance database. The prediction errors calculated for all reflectances in the database are arranged in ascending order, and only a certain number of spectral reflectances from the beginning are selected as the local optimal candidates for spectral reflectance estimation.

Section 4 describes the method used to determine the best reflectance estimate using the local optimal dataset. The Wiener and LMMSE estimators are proposed as first-class statistical methods based on a limited number of local optimal reflectance data. Linear programming and quadratic programming methods are proposed as second-class methods for solving optimization problems under constraints imposed.

Section 5 presents the experiments performed to validate the proposed methods for estimating surface-spectral reflectance. Different mobile phone cameras, LED light sources, a standard spectral reflectance database, and standard test samples are used in those experiments. The performances of the proposed methods are examined in detail and compared with those of other methods.

## 2. Observation Model

**Figure 1:**
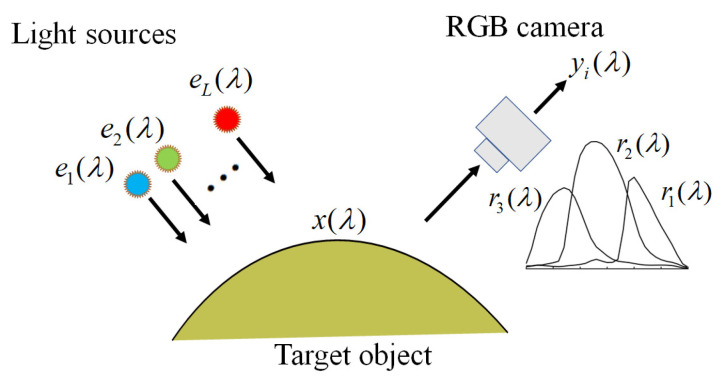
Schematic diagram of our multispectral image acquisition system.

The observation model for our multispectral image acquisition system is depicted schematically in Figure 1. It is constructed using an RGB camera with three color channels (*c* = 1, 2, 3) and multiple light sources with *L* different illuminant spectra (*l* = 1, 2, ..., *L*). Therefore, we obtain *m* = 3 *L* observations for a single target object. Because a linear relationship exists between the camera responses and surface-spectral reflectance (see [27]), we express the observations $y_{i}$ as

(1) $$y_{i}\;=\;g\int_{400}^{700}x(\lambda )e_{l}(\lambda )r_{c}(\lambda )d\lambda \;\;+\;n_{i},\;(i=1,\;2,\;\ldots ,\;m),$$

where x(λ) is the surface-spectral reflectance of the target object, $e_{l}(\lambda )$ (*l* = 1, 2, …, *L*) represents the spectral power distribution of the light sources, $r_{c}(\lambda )$ and (*c* = 1, 2, 3) denotes the spectral sensitivity functions of the camera. The wavelength λ is in the visible range of 400–700 nm. The additive noise $n_{i}$ in the imaging system is assumed to be white noise with zero mean and variance *a* and is uncorrelated with x(λ). Here, $y_{i}\;$ represents the digital camera outputs, while x(λ), $e_{l}(\lambda )$, and $r_{c}(\lambda )$ are physical quantities.

The coefficient g in Equation (1) is the weight used to convert the model outputs to the practical digital outputs, called the *gain parameter*. The parameter g is unique to the imaging system and depends on the conditions of the imaging system, such as the locations of the camera and light sources, including illumination intensities. How to determine the noise variance *a* and the gain parameter *g* was shown in [13].

The spectral functions of reflectance, illuminants, and sensitivities are sampled at *n* wavelength points with equal intervals in the range of 400–700 nm and described using *n*-dimensional column vectors as follows:

(2) $$x=\left[\begin{array}{c} x(\lambda_{1})\\ x(\lambda_{2})\\ \vdots \\ x(\lambda_{n}) \end{array}\right],\;\;\;\;\;\;e_{l}=\left[\begin{array}{c} e_{l}(\lambda_{1})\\ e_{l}(\lambda_{2})\\ \vdots \\ e_{l}(\lambda_{n}) \end{array}\right],\;\;\;\;\;\;r_{c}=\left[\begin{array}{c} r_{c}(\lambda_{1})\\ r_{c}(\lambda_{2})\\ \vdots \\ r_{c}(\lambda_{n}) \end{array}\right],$$

where *l* = 1, 2, …, *L* and *c* = 1, 2, 3. The discrete representation of the observation model is expressed as follows:

(3) y = gAx + n,

where

(4) $$y=\;\left[\begin{array}{c} y_{1}\\ y_{2}\\ \vdots \\ y_{m} \end{array}\right],\;\;\;\;A=\;\left[\begin{array}{c} {\left(e_{1}.*r_{1}\right)}^{t}\Delta \lambda \\ {\left(e_{2}.*r_{2}\right)}^{t}\Delta \lambda \\ \vdots \\ {\left(e_{L}.*r_{3}\right)}^{t}\Delta \lambda \end{array}\right],\;\;\;\;n\;=\;\left[\begin{array}{c} n_{1}\\ n_{2}\\ \vdots \\ n_{m} \end{array}\right]$$

The symbol (.*), superscript *t*, and Δλ represent elementwise multiplication, matrix transposition, and the wavelength sampling interval, respectively. Therefore, **A** is an (*m × n*) matrix defined by the illuminant spectra and the spectral sensitivities, and **n** is an *n*-dimensional noise vector.

## 3. Selection of Local Optimal Reflectance Dataset

Previously, adaptive Wiener estimation methods were proposed to improve the estimation accuracy of spectral reflectance, where training samples were adaptively selected to perform autocorrelation matrix calculation in the Wiener estimator [7,11]. Although the original Wiener estimator uses all data of a spectral reflectance database, the basic idea of the adaptive method is as follows: if the training reflectance samples for calculating the autocorrelation matrix are close to the target reflectance and close to each other, then the estimation may be reliable. Therefore, first, the spectral reflectance estimate ${\hat{x}}_{\mathrm{all}}$ is calculated from observation **y** using the original Wiener estimator. Second, the spectral similarity or distance ${\left\|{\hat{x}}_{\mathrm{all}}-x_{i}\right\|}^{2}$ is calculated between the estimated spectral reflectance ${\hat{x}}_{\mathrm{all}}$ and each spectral reflectance $x_{i}$ in the original database. Third, a set of spectral reflectances $x_{i}$ is selected based on the similarity or distance from the reflectance database. However, we should note that because the estimate ${\hat{x}}_{\mathrm{all}}$ obtained in the first step is contaminated with the estimation error, the most similar reflectances or the reflectances with the shortest distance to ${\hat{x}}_{\mathrm{all}}$ are not necessarily the best estimates.

**Figure 2:**
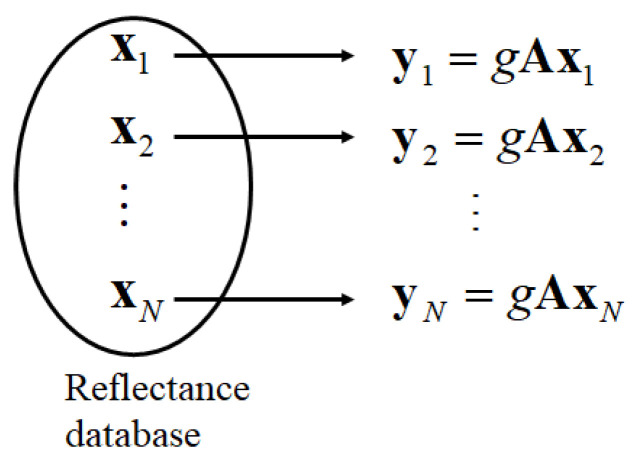
Prediction of observation for each spectral reflectance in the database.

Herein, we propose a more direct method for selecting a local optimal reflectance dataset. Let *N* be the number of spectral reflectances in the database. First, we predict the observations using Equation (3) in the form of $gAx_{i}^{}$ for each spectral reflectance $x_{i}$ (*i* = 1, 2, ..., *N*) in the database, as shown in Figure 2. Second, we calculate the prediction error for observation **y** as follows:

(5) $$L_{i}={\left\|y-gAx_{i}^{}\right\|}_{2}^{2}\;(i=1,\;2,\;...,\;N),\;$$

where the norm ${\left\|•\right\|}_{2}^{2}$ is defined as ${\left\|z\right\|}_{2}^{2}=z_{1}^{2}+z_{2}^{2}+\ldots +z_{m}^{2}$. Third, the prediction errors are arranged in the ascending order as $L_{\left(1\right)}\leq L_{\left(2\right)}\leq \cdots \leq L_{\left(N\right)}$, and the corresponding spectral reflectances are $x_{\left(1\right)}$, $x_{\left(2\right)}$, …, $x_{\left(N\right)}$. Finally, the first *K* spectral reflectances $x_{\left(1\right)}$, $x_{\left(2\right)}$, …, $x_{\left(K\right)}$ are selected as the local optimal candidates to estimate the spectral reflectance. The superiority of this method is demonstrated in subsequent experiments.

## 4. Determination of Reflectance Estimate Using Local Optimal Dataset

The best spectral reflectance estimate can be obtained using only the local optimal reflectance dataset. We herein propose four algorithms that can yield the best estimates, which minimize the estimation error. The Wiener estimator and LMMSE estimator are used as first-class statistical methods based on a limited number of local optimal reflectance data. Linear programming and quadratic programming are applied as second-class methods for solving optimization problems under constraints imposed.

### 4.1. Local Wiener Method

The estimate of **x**, $\hat{x}$, is written in the form $\hat{x}\;=\;By,$ where **B** is an (*n × m*) matrix. The matrix **B** is determined to minimize the mean-square error (MSE) between the estimate $\hat{x}$ and original **x**, which is defined as

(6) $$J\;=\;E\left[{\left\|x\;-\;\hat{x}\right\|}_{2}^{2}\right]=\;\mathrm{tr}\left(E\left[(x\;-\;\hat{x}){\left(x\;-\;\hat{x}\right)}^{t}\right]\right),$$

where the expectation operator **E**[**x**] denotes the mean or average of **x**, and tr(**X**) represents the trace of a square matrix **X**. Consequently, the estimate is solved as

(7) $$\hat{x}\;=\;gRA^{t}{\left(g^{2}ARA^{t}+aI\right)}^{-1}y,$$

where **R** is the autocorrelation matrix $E\left[xx^{t}\right]\;$, and **I** is the (*n × n*) identity matrix, with 1’s in the diagonal and 0’s elsewhere.

Although the autocorrelation matrix is typically calculated using all available datasets of various spectral reflectances, in this study, **R** is calculated using only the *K* local optimal reflectances. When we define an (*n × K*) matrix **X** as

(8) $$X=\left[\begin{array}{cccc} x_{\left(1\right)} & x_{\left(2\right)} & \cdots & x_{\left(K\right)} \end{array}\right]$$

we have

(9) $$R=XX^{t}/K$$

The autocorrelation matrix can be more statistically precise compared with using a dataset that includes many other reflectances.

### 4.2. Local LMMSE Method

In LMMSE estimation, the estimate $\hat{x}$ is determined in the more general form $\hat{x}\;=\;By\;+b,$ where **b** is an *n*-dimensional constant vector. Because the average surface-spectral reflectance is not zero, we set the averages of **x** and **y** as $E\left[x\right]=x_{0}$ and $E\left[y\right]=y_{0}=gAx_{0}$, respectively, and the optimal estimate to minimize the MSE is expressed as

(10) $$\hat{x}\;=\;x_{0}\;+\;gPA^{t}{\left(g^{2}APA^{t}\;+\;aI\right)}^{-1}\left(y-gAx_{0}\right),$$

where **P** is the covariance matrix of **x**, defined as $P=E\left[\left(x-x_{0}\right){\left(x-x_{0}\right)}^{t}\right]$ [13]. When we compare the estimation error $J_{2}$ of the LMMSE estimator to the error $J_{1}$ of the Wiener estimator, a clear relationship exists, i.e., $J_{1}\geq J_{2}$ (see [13]). In other words, the estimation accuracy of the LMMSE estimator always exceeds that of the Wiener estimator.

In this study, the average of surface-spectra reflectance is calculated as

(11) $$x_{0}=\sum_{i=1}^{K}x_{\left(i\right)}/K$$

The covariance matrix is calculated using only the *K* local optimal reflectances and the average as

(12) $$P=R-x_{0}x_{0}^{t}$$

Notably, because the covariance matrix is approximated using a small number of spectral reflectances, the relationship $J_{1}\geq J_{2}$ does not necessarily hold.

### 4.3. Linear Programming Method

The spectral reflectance is estimated in a linear combination of *K* local optimal data as

(13) $$\hat{x}\;=\;\alpha_{1}x_{\left(1\right)}\;+\;\alpha_{2}x_{\left(2\right)}\;+\;\cdots \;+\alpha_{K}x_{\left(K\right)},$$

where $\alpha_{i}$ (*i* = 1, 2, …, *K*) are weighting coefficients. Let ${\left(\hat{x}\right)}_{j}$ be the *j*-th element of the estimate,

(14) $${\left(\hat{x}\right)}_{j}\;=\;\alpha_{1}{\left(x_{\left(1\right)}\right)}_{j}\;+\;\alpha_{2}{\left(x_{\left(2\right)}\right)}_{j}\;+\;\cdots \;+\alpha_{K}{\left(x_{\left(K\right)}\right)}_{j}.$$

Because of the physical constraint of spectral reflectance, we have

(15) $$0\leq {\left(x_{\left(i\right)}\right)}_{j}\;\leq \;1.\;(i=1,\;2,\;\ldots ,\;K)\;(j=1,\;2,\;\ldots ,\;n)$$

As a sufficient condition to satisfy this constraint, we define a constraint on $\alpha_{i}$ as follows:

(16) $$\sum_{i=1}^{K}\alpha_{i}=1,\;\alpha_{i}\geq 0.\;(i=1,\;2,\;\ldots ,\;K)$$

Next, we consider the L_1_-norm minimization problem as follows:

(17) $${\left\|y-F\alpha \right\|}_{1}\;=\;\sum_{i=1}^{m}\left|y_{i}\;-\;{\left(F\alpha \right)}_{i}\right|\;\;\;\;\;\;\;\;=>\;\min,$$

where

(18) F=gAX,

(19) $$\alpha ={\left[\begin{array}{cccc} \alpha_{1} & \alpha_{2} & \cdots & \alpha_{K} \end{array}\right]}^{t}.$$

This minimization is equivalent to the following linear programming problem (see [32]):

(20) $$\min\left\{\sum_{i=1}^{m}u_{i}\right\}\;\mathrm{subject}\;\mathrm{to}-u_{i}\leq y_{i}\;-\;{\left(F\alpha \right)}_{i}\leq u_{i}.\;(i=1,\;2,\;\ldots ,\;m),$$

In addition, when we define a *K*-dimensional column vector **p** with all elements equal to 1, we can rewrite the constraint on the weights in Equation (16) as $p^{t}\alpha =1$, which is equivalent to $p^{t}\alpha \leq 1$ and $-p^{t}\alpha \leq -1$. The constraint of $\alpha_{i}\geq 0$ (*i* = 1, 2, …, *K*) is expressed as $-l_{K}\alpha \leq 0$, where $l_{K}$ demotes a (*K* × *K*) identity matrix. Let **u** be an (*m* × 1) vector whose *i*-th component is $u_{i}$.

Therefore, the linear programming problem above can be summarized as

(21) $$\min\left\{\left[0\;0\;\cdots 0\;1\;1\;\cdots 1\right]\left[\begin{array}{c} \alpha \\ u \end{array}\right]\right\}\;\mathrm{subject}\;\mathrm{to}\;\;\;\;\;\left[\begin{array}{cc} F & -l_{m}\\ -F & -l_{m}\\ -l_{K} & 0\\ p^{t} & 0\\ -p^{t} & 0 \end{array}\right]\left[\begin{array}{c} \alpha \\ u \end{array}\right]\leq \left[\begin{array}{c} y\\ -y\\ 0\\ 1\\ -1 \end{array}\right],$$

where $\left[0\;0\;\cdots 0\;1\;1\;\cdots 1\right]$ denotes a (1 × (*K* + *m*)) matrix comprising *K* 0’s and *m* 1’s, and $l_{m}$ denotes an (*m* × *m*) identity matrix. The MATLAB function “linprog” is available for solving the present problem [33]. To utilize this function, we introduce the following matrices:

(22) $$H=\left[\begin{array}{cc} F & -l_{m}\\ -F & -l_{m}\\ -l_{k} & 0\\ p^{t} & 0\\ -p^{t} & 0 \end{array}\right],\;\;\;\;\;\;\;\;h=\left[\begin{array}{c} y\\ -y\\ 0\\ 1\\ -1 \end{array}\right],\;\;\;\;\;\;\;\;f=\left[\begin{array}{c} 0\\ 0\\ \vdots \\ 0\\ 1\\ 1\\ \vdots \\ 1 \end{array}\right].$$

Subsequently, we can use z=linprog(f, H, h) to solve the linear programming problem. The solution is given as

(23) $$z={\left[\begin{array}{cccccccc} {\hat{\alpha }}_{1} & {\hat{\alpha }}_{2} & \cdots & {\hat{\alpha }}_{K} & {\hat{u}}_{1} & {\hat{u}}_{2} & \cdots & {\hat{u}}_{m} \end{array}\right]}^{t},$$

where $\hat{\alpha }={\left[\begin{array}{cccc} {\hat{\alpha }}_{1} & {\hat{\alpha }}_{2} & \cdots & {\hat{\alpha }}_{K} \end{array}\right]}^{t}$ is the optimal estimate of the weights. The spectral reflectance estimate is written as $\hat{x}\;=X\hat{\alpha }$.

### 4.4. Quadratic Programming Method

Next, we consider the following L_2_-norm minimization problem:

(24) $${\left\|y-F\alpha \right\|}_{2}^{2}\;\;\;\;\;\;\;=>\;\min,$$

where **F** and α are defined in Equations (18) and (19), respectively. Quadratic programming is the optimization problem of identifying a vector **z** that minimizes a quadratic function

(25) $$\min\left\{\frac{1}{2}z^{t}Hz+f^{t}z\right\}\;\;\;\;\;\;\;\;\;\mathrm{subject}\;\mathrm{to}\;\mathrm{constraint}$$

$$Dz\leq d\;\;\;\;(\mathrm{inequality}\;\mathrm{constraint})\;\;\mathrm{or}\;\;D_{\mathrm{eq}}z=d_{\mathrm{eq}}\;\;\;\;(\mathrm{equality}\;\mathrm{constraint}).$$

The current L_2_-norm minimization problem can be rewritten to fit the quadratic programming problem as follows:

(26) $$\min\left\{\frac{1}{2}\alpha^{t}F^{t}F\alpha -{\left(F^{t}y\right)}^{t}\alpha \right\}\;\;\;\;\;\;\;\;\;\mathrm{subject}\;\mathrm{to}\;\;\alpha \geq 0,\;\;p^{t}\alpha =1.$$

The MATLAB function “quadprog” is available for solving the present problem [34]. To utilize this function, we define the function arguments as

(27) $$z=\alpha ,\;H=F^{t}F,\;f=-F^{t}y,\;D=-I_{K},\;d=0,\;D_{\mathrm{eq}}=p^{t},\;d_{\mathrm{eq}}=1.$$

Then, $z=\mathrm{quadprog}(H,f,\;D,\;d,\;D_{\mathrm{eq}},\;d_{\mathrm{eq}})$ solves the quadratic programming problem. The solution is $z=\hat{\alpha }$, and the spectral reflectance estimate is written as $\hat{x}\;=X\hat{\alpha }$.

## 5. Overall Flow of Estimation Procedure

**Figure 3:**
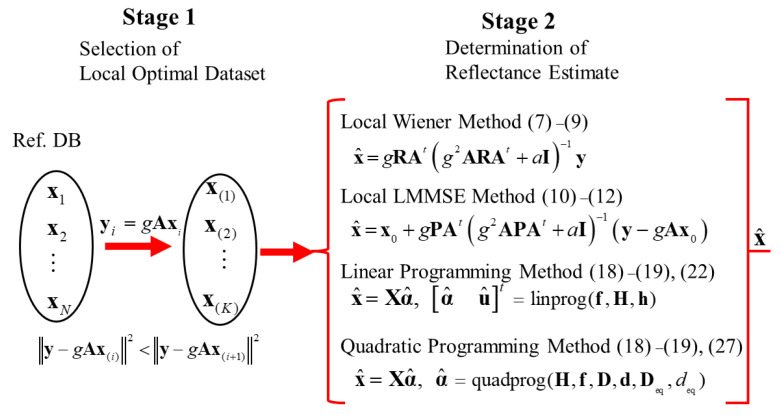
Overall flow of the proposed methods for estimating spectral reflectance in two stages.

Figure 3 depicts the overall flow of the proposed methods for estimating spectral reflectance in two stages, where the first stage is to select the local optimal dataset suitable for the observed camera outputs from the original reflectance database and the second stage is to determine the reflectance estimate using the local optimal dataset only.

## 6. Experimental Results

### 6.1. Experimental Setup

**Figure 4:**
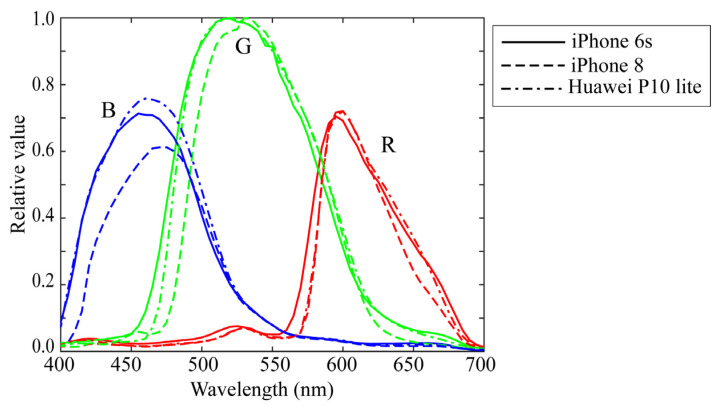
Relative RGB spectral sensitivity functions of three mobile phone cameras, where red, green, and blue curves correspond to Apple iPhone 6s, Apple iPhone 8, and Huawei P10 lite cameras, respectively.

Experiments were conducted to validate the superiority of the proposed method for estimating surface-spectral reflectance from image data. We used different mobile phone cameras, LED light sources, a standard spectral reflectance database, and standard test samples. Three mobile phone cameras selected from iOS and Android phone cameras were (1) Apple iPhone 6s (Protek, Shanghai, China), (2) Apple iPhone 8 (Protek, Shanghai, China)), and (3) Huawei P10 lite (Huawei, Shenzhen, China). Figure 4 shows the relative RGB spectral sensitivity functions, where the solid, broken, and dash-dot curves correspond to curves correspond to (1), (2), and (3), respectively. The numerical data of the spectral sensitivity functions are available at http://ohlab.kic.ac.jp/ (accessed on 10 January 2023). The camera images were captured in a lossless raw image format in Adobe digital negative format. The dark response was measured on all selected cameras and discarded from the camera output. The depth of the cameras employed was 12 bits.

**Figure 5:**
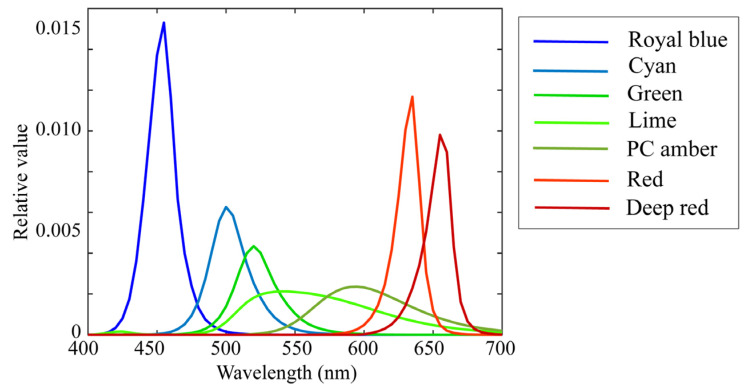
Spectral power distributions of seven LED light sources used in current experiments.

The illumination light sources were seven (*L* = 7) LED light sources, the spectral power distributions of which are shown in Figure 5. The standard spectral reflectance database used in this paper is available at http://ohlab.kic.ac.jp/ (accessed on 10 January 2023), which is a dataset of 1776 (=*N*) spectral reflectances and comprised of Dupont spectral data, Munsell spectral data, and various object spectral data, including man-made objects and natural objects. All spectral curves were sampled at 61 (=*n*) points with 5-nm intervals in the visible range of 400–700 nm and represented by 61-dimensional column vectors. The X-Rite Color Checker Passport Photo (X-Rite, Grand Rapids, MI, USA) was used as the standard test target to validate reflectance estimation. This target comprised 24 color checkers whose spectral reflectance values were measured using a spectral colorimeter.

Spectralon (Labsphere, North Sutton, NH, USA) was used as a white reference standard and was placed near the target samples. The cameras were placed close to the target, and the light sources were placed a little apart from the target. These positions are the same as in the previous paper. [13]. The parameters *g* and *a* of the gain and noise variance in the observation model, respectively, were determined using the calibration method in [13] based on the Spectralon data and L1-norm minimization.

### 6.2. Performances of Proposed Methods

**Figure 6:**
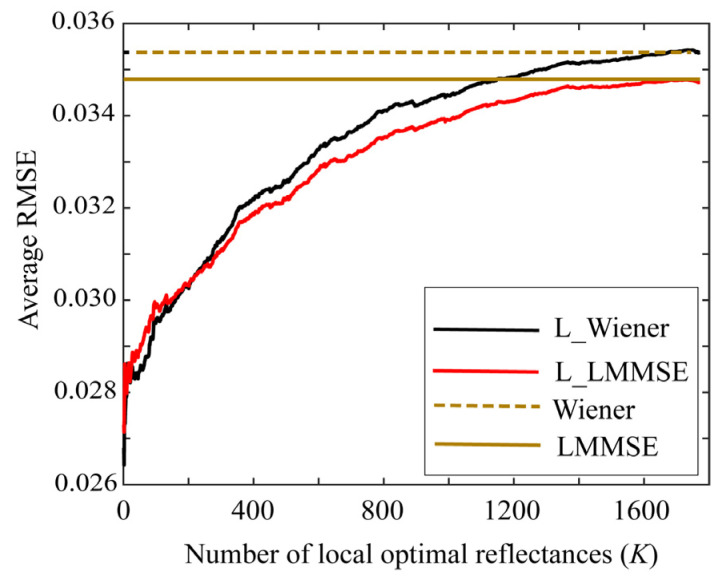
Average RMSEs of the proposed Wiener and LMMSE methods for 24 color checkers as a function of number *K* of local optimal reflectances when the methods are applied to the image data using Apple iPhone 6s.

First, we examined the performances of the proposed Wiener and LMMSE methods using the local optimal reflectance dataset. Figure 6 shows the average root-mean-square errors (RMSEs) for the 24 color checkers as a function of the number *K* of the local optimal reflectances when the two methods were applied to the image data using the Apple iPhone 6s. In the figure, “L_Wiener” and “L_LMMSE” represent the local Wiener and LMMSE methods using the local optimal dataset in Section 4.1 and Section 4.2, respectively, and “Wiener” and “LMMSE” represent the respective original methods using all data. Because the original methods use all the spectral reflectances in the database, the average RMSEs are constant values of 0.03537 and 0.03479 for the Wiener and LMMSE methods, respectively, independently of *K*. The average RMSE is calculated as the root of the average of the squared norm of the estimation error per wavelength over the 24 color checkers, i.e.,

(28) $$E[\mathrm{RMSE}]={\left\{\left(\sum_{i=1}^{24}{\left\|x_{i}-\;{\hat{x}}_{i}\right\|}^{2}/61\right)/24\right\}}^{1/2}$$

This calculation was changed from the calculation presented in [13].

Figure 6 clearly suggests that the estimation accuracies of L_Wiener and L_LMMSE approach the accuracies of the original methods as the number *K* of the local optimal reflectances used in the reflectance estimation increases. Based on our observations, the estimation improves as *K* decreases, as compared with the case in the original methods. In other words, the proposed methods are more effective for smaller *K* values. The results yielded by the Apple iPhone 6s are similar to those yielded by the mobile phone cameras, Apple iPhone 8, and Huawei P10 lite.

**Figure 7:**
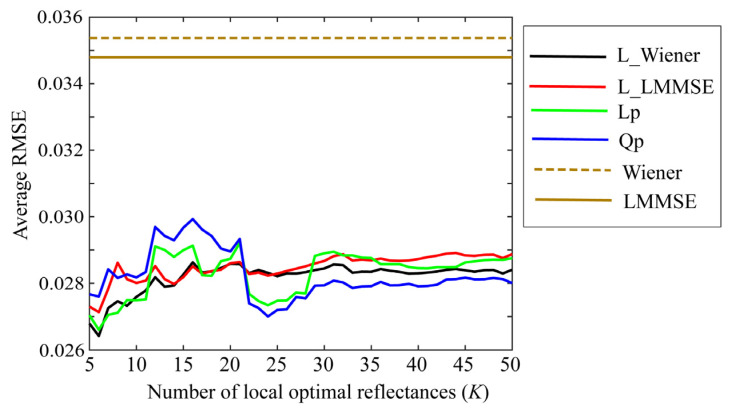
Average RMSEs obtained by the proposed methods for 24 color checkers in the range of small *K* of 5–50 when applying the methods to image data acquired using Apple iPhone 6s.

**Figure 8:**
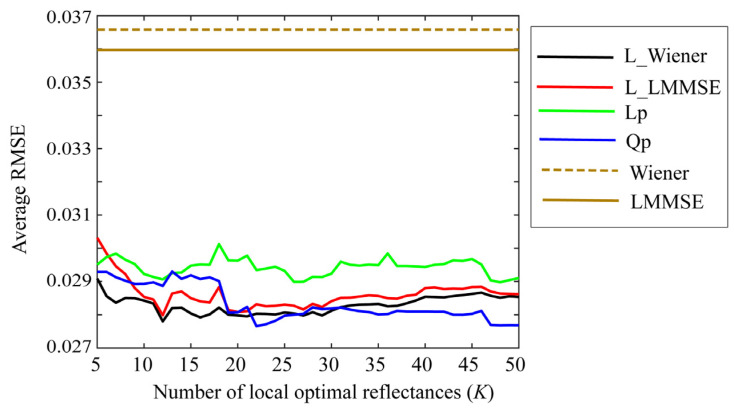
Average RMSEs obtained by the proposed methods for 24 color checkers in the range of small *K* of 5–50 when applying the methods to image data acquired using Apple iPhone 8.

**Figure 9:**
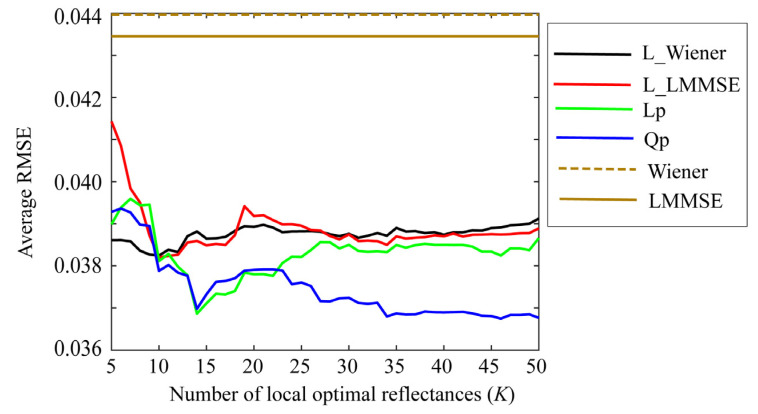
Average RMSEs obtained by the proposed methods for 24 color checkers in the range of small *K* of 5–50 when applying the methods to image data acquired using Huawei P10 lite.

Small values of *K* are preferred to simplify the estimation. Figure 7, Figure 8 and Figure 9 depict the average RMSEs yielded by each of the four proposed methods for the 24-color checkers in the range of small *K* of 5–50, when applying the methods to the image data acquired using the Apple iPhone 6s, Apple iPhone 8, and Huawei P10 lite. The symbols Lp and Qp denote the linear programming and quadratic programming methods in Section 4.3 and Section 4.4, respectively. As shown in each figure depicting the results of the mobile phone cameras, all the proposed methods in this range are significantly more accurate than the original Wiener and LMMSE methods. We disregarded the performance curves of the average RMSEs in the range of *K* < 5 owing to their instability. Among the proposed methods, the best one cannot be determined within this range. However, the quadratic programming method appears to be significantly better than the others, as its error in the large range of *K* for all cameras used remains low. As can be seen from Figure 7, Figure 8 and Figure 9, the average RMSEs using Huawei P10 lite are significantly worse. We investigated this point, and found that, although the first part in the local optimal reflectance dataset $x_{\left(1\right)}$, $x_{\left(2\right)}$, ..., $x_{\left(K\right)}$ was the same for both Apple iPhones 6s and 8, the corresponding part selected for Huawei P10 lite was different from iPhones. This difference in the selected optimal dataset was reflected in the estimation results.

**Figure 10:**
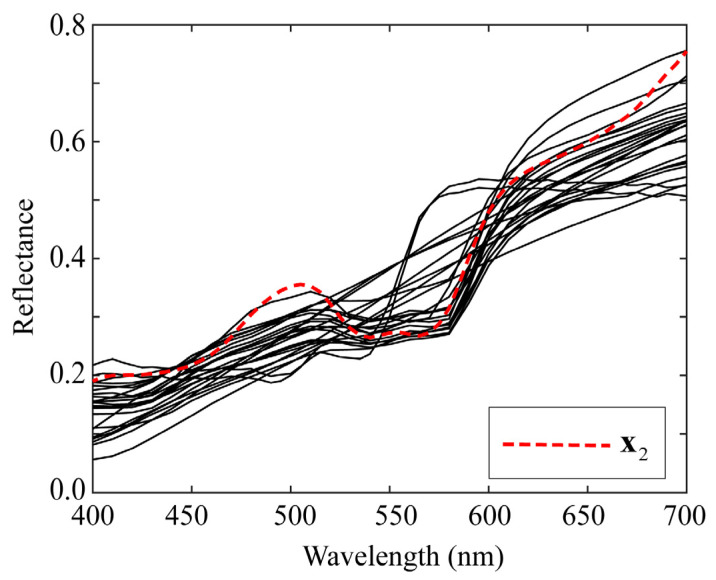
Spectral curves of 23 local optimal reflectances used for estimating second spectral reflectance $x_{2}$ in 24 color checkers.

**Figure 11:**
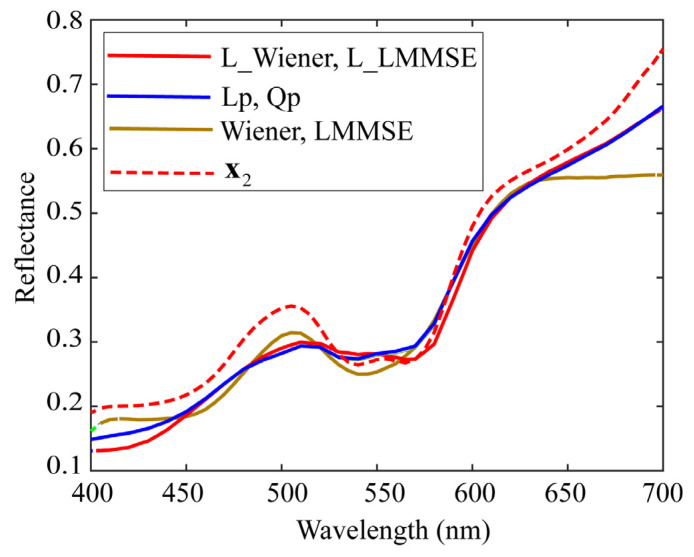
Spectral curves estimated using the four proposed methods for spectral reflectance $x_{2}$.

Next, we comprehensively examined the estimation results for a specific spectral reflectance. The second patch among the color checkers represents light skin. We choose *K* = 23 as a representative value of *K* in the range 5–50. Suppose we use the image data of the iPhone 6s. Figure 10 and Figure 11 show the estimation results for the second spectral reflectance for the 24 color checkers. The spectral curves of the 23 local optimal reflectances are shown in Figure 10, where the broken red curve represents the measured spectral reflectance $x_{2}$. A set of spectral reflectances close to $x_{2}$ was selected from the database as the local optimal dataset. The spectral curves estimated using the four proposed methods for $x_{2}$ are depicted in Figure 11, where the brown curves represent the spectral reflectances estimated via the original Wiener and LMMSE methods, and the two curves are overlapped and visually indistinguishable; the red curves represent the two estimates yielded by the proposed L_Wiener and L_LMMSE methods, and the blue curves represent the two estimates yielded by the proposed linear and quadratic programming methods based on the local optimal reflectances shown in Figure 10. In this case, the quadratic programming method achieved the best performance, although the estimated reflectance presented a spectral curve similar to that presented by the linear programming method.

Furthermore, it should be noted that the proposed methods based on the local optimal dataset increase obviously in computation time, in comparison with the direct Wiener and LMMSE methods based on the original dataset. All computations in experiments were executed using MATLAB (R2022b) on the PC of Panasonic CF-LX6 (Panasonic, Osaka, Japan). The average computation time for the direct Wiener and LMMSE methods was about 0.0045 s. Also, the computation time was compared using the L_Wiener method, L_LMMSE method, linear programming method, and quadratic programming method. The average computation times when using *K* = 23 local optimal reflectances were 0.1958, 0.1932, 0.5325, and 0.3425 s, respectively. The determination of the local optimal dataset in the first stage took a lot of time.

### 6.3. Comparisons with Other Methods

#### 6.3.1. Local Optimal Reflectance Dataset

We changed the selection of a local optimal reflectance dataset. Although we here proposed using the prediction error $\left(y-gAx_{i}\right)$ for the observation, the previous adaptive Wiener estimation methods were based on the estimation error $\left({\hat{x}}_{\mathrm{all}}-x_{i}\right)$ [7,11]. The estimate ${\hat{x}}_{\mathrm{all}}$ was calculated using the original Wiener estimator based on all the spectral reflectances in the database. Subsequently, the local optimal spectral reflectances were selected based on (1) a similarity measure, e.g., the spectral angle, or (2) a distance measure, e.g., the MSE.

First, we examined the spectral angle, which represents the spectral similarity between two vectors in a high-dimensional spectral space. In our case, the spectral angle θ is defined as $\theta_{i}=\cos^{-1}\left({\hat{x}}_{\mathrm{all}}^{t}x_{i}/\left(\left\|{\hat{x}}_{\mathrm{all}}\right\|\left\|x_{i}\right\|\right)\right)$ (*i* = 1, 2, …, *N*). As $\theta_{i}$ decreases, the two vectors ${\hat{x}}_{\mathrm{all}}$ and $x_{i}$ become increasingly similar. The angles $\theta_{i}$ for all reflectances were arranged in descending order, and the first *K* spectral reflectances were selected as the locally optimal dataset. However, the average RMSEs were greater than the error of the original Wiener estimator; in other words, the spectral angle did not yield the expected result.

**Figure 12:**
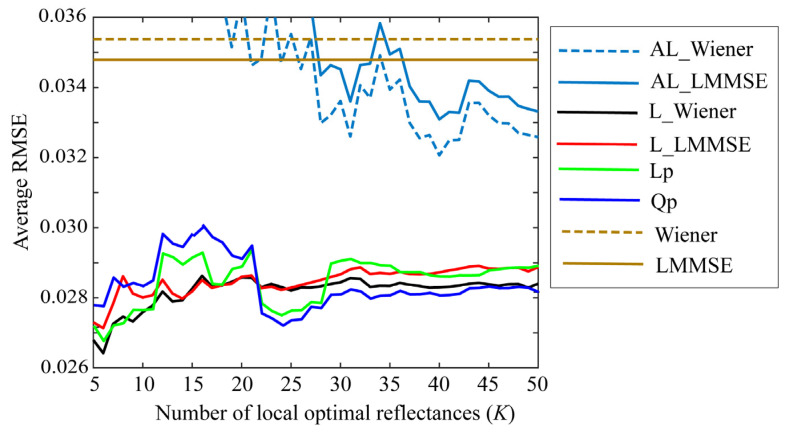
Average RMSEs yielded by the previous method compared with results obtained using the proposed method shown in Figure 6, when using iPhone 6s. Bold and broken cyan curves represent the previous method based on ${\left\|{\hat{x}}_{\mathrm{all}}-x_{i}\right\|}_{2}^{2}$; AL_Wiener (AL_LMMSE) represents the adaptive local Wiener (LMMSE), where ${\hat{x}}_{\mathrm{all}}$ was calculated using the original Wiener (LMMSE), and then the best estimates $\hat{x}$ based on the selected dataset, L_Wiener, L_LMMSE, Lp, and Qp were calculated using the methods in Section 4.1, Section 4.2, Section 4.3 and Section 4.4.

Second, we examined the MSE ${\left\|{\hat{x}}_{\mathrm{all}}-x_{i}\right\|}_{2}^{2}$, (*i* = 1, 2, …, *N*). We calculated the estimate ${\hat{x}}_{\mathrm{all}}$ by the Wiener and LMMSE estimators using all the reflectance data. The MSEs for all reflectances were arranged in ascending order, and the first *K* spectral reflectances were selected. Figure 12 shows a comparison of the average RMSEs for the 24-color checkers along with the results yielded by the proposed method shown in Figure 7, when using the iPhone 6s. In Figure 12, AL_Wiener (AL_LMMSE) on the cyan curve represents the adaptive local Wiener (LMMSE), where ${\hat{x}}_{\mathrm{all}}$ was calculated using the original Wiener (LMMSE), and then the best estimates $\hat{x}$ based on the selected dataset, L_Wiener, L_LMMSE, Lp, and Qp were calculated using the methods in Section 4.1, Section 4.2, Section 4.3 and Section 4.4. Thus, the proposed method for selecting the local optimal reflectances based on the prediction error ${\left\|y-gAx_{i}\right\|}_{2}^{2}$ of the observation is superior to the previous method related to ${\left\|{\hat{x}}_{\mathrm{all}}-x_{i}\right\|}_{2}^{2}$.

#### 6.3.2. Single RGB-Based Spectral Estimation

Spectral reflectance estimation was often performed using RGB image data from a single-color camera instead of a multispectral imaging system, particularly in the training-based approach. This is because the imaging system is easy to use without modification and eliminates the need for camera spectral sensitivity functions. A broadband light source was preferred in the single RGB imaging system.

Typical methods for efficiently determining reflectance estimates from local optimal training samples were (1) nonlinear expansion of RGB data and (2) introduction of weighting factors (see [16,22]). For (1), consider an example that [*r g b*] is expanded to $\left[1\;\;r\;\;g\;\;b\;\;r^{2}\;\;g^{2}\;\;b^{2}\;\;rg\;\;gb\;\;br\right]$. Our experimental results suggested that the nonlinear expansion method did not perform well because of its instability. For (2), consider an example where inverse distances such as ${1/\left({\left\|y-y_{i}\right\|}_{2}^{2}\right)}^{1/2}$ (*i* = 1, 2, …, *K*) are applied to the local optimal reflectances as weighting coefficients.

**Figure 13:**
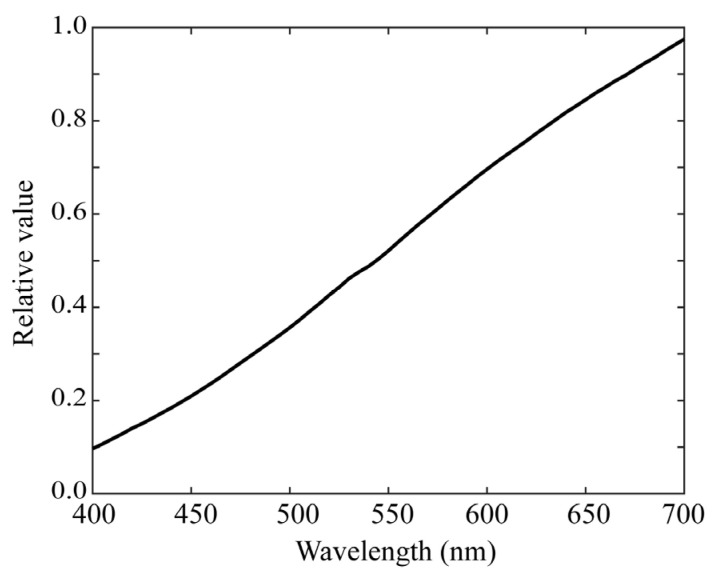
Spectral power distribution of the incandescent lamp used in an experiment on the single RGB-based spectral estimation.

**Figure 14:**
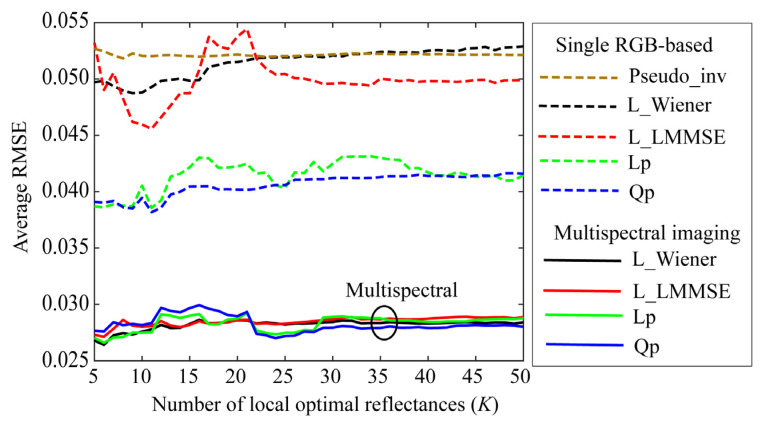
Reflectance estimation results for single RGB-based spectral estimation, where RGB images for 24 color checkers were acquired using iPhone 6s. Pseudo_inv represents the average RMSEs yielded by the pseudo-inverse method using the weights as a function of the number of the selected local optimal reflectances. L_Wiener, L_MMSE, Lp, and Qp denote the proposed methods using the local optimal dataset without using the weights, respectively. Four spectral curves at bottom of the figure show the average RMSEs in Figure 7 reconstructed using a multispectral imaging system for comparison.

We performed experiments for the single RGB-based spectral estimation based on method (2), where an incandescent lamp with the spectral power distribution shown in Figure 13 was used as a broadband light source. The experimental results are shown in Figure 14, where the RGB images for the 24-color checkers were acquired using the iPhone 6s. Because the camera spectral sensitivities were known in our case, we first predicted the camera outputs for each of the training reflectance samples as $y_{i}=gAx_{i}$ without capturing actual images; subsequently, we calculated an (*n* × *K*) reflectance matrix **X** and a (3 × *K*) observation matrix **Y** in the forms $X=\left[\begin{array}{cccc} x_{\left(1\right)} & x_{\left(2\right)} & \cdots & x_{\left(K\right)} \end{array}\right]$ and Y=gAX. We defined a (*K* × *K*) weighting matrix as

(29) $$W=\mathrm{diagonal}\left(\begin{array}{cccc} w_{11} & w_{22} & \cdots & w_{KK} \end{array}\right),$$

where

(30) $$w_{ii}={1/\left({\left\|y-y_{i}\right\|}_{2}^{2}\right)}^{1/2}\;(i=1,\;2,\;\ldots ,\;K).\;$$

Based on the previous pseudo-inverse method (see [16,22]), the estimate $\hat{x}$ based on the local optimal dataset was calculated as

(31) $$\hat{x}=Qy,$$

where

(32) $$Q=XW{\left(YW\right)}^{+}.$$

The symbol + denotes the pseudo-inverse of a matrix.

The broken brown curve with Pseudo_inv in Figure 14 shows the average RMSEs obtained using the pseudo-inverse method as a function of the number of selected local optimal reflectances. The accuracy is similar to that of L_Wiener without using the weights presented in Section 4. A. In the figure, L_MMSE, Lp, and Qp denote the proposed LMMSE, linear programming, and quadratic programming methods for the single RGB images without using weights, respectively. In addition, the estimation accuracies of the linear and quadratic programming methods exceed those of L_Wiener and L_LMMSE.

The four bold curves at the bottom of Figure 14 depict the average RMSEs in Figure 7 reconstructed using the multispectral imaging system for comparison. The multispectral imaging system is clearly superior to the single RGB imaging system. From the viewpoint of the estimation procedure, we should note that the statistical methods require the variance of the system noise, whereas mathematical programming methods do not require such statistical knowledge.

Using the single-exposure method, there are other methods than using the local optimal dataset. Here, we applied two methods to the present problem and examined the performances; one was the interpolation method [35] and another was the weighted principal component analysis (PCA) method [36]. In the interpolation method, all spectral reflectances in the original database $x_{i}$ (*i* = 1, 2, …, *N*) were first mapped into the three-dimensional RGB space by $y_{i}=gAx_{i}$, where four vertices with the RGB values defined a tetrahedron and the RGB space was partitioned by many different tetrahedrons. Next, we found a tetrahedron containing the observation **y** and estimated the RGB coordinates of **y** using the four vertices. This linear interpolation was applied to the corresponding four spectral reflectances in the database to obtain the reflectance estimate. The MATLAB function “delaunayTriangulation” was used for the tetrahedrization procedure.

In the weighted PCA method, the spectra reflectances were represented as the weighted sum of three orthogonal basis functions. The basis functions are called the principal component vectors, which are obtained by the singular value decomposition of the original reflectance data matrix or the eigendecomposition of the data covariance matrix. We used the same weights as Equation (30) and the (*N* × *N*) weighting matrix **W** in Equation (29) was applied to the original data matrix.

In experimental results for the interpolation method, we could not find suitable tetrahedrons for the 17th (green) and 19th (white) patches among the X-rite color checkers. This was because the coordinates of these patches were out of the gamut determined by the original reflectance database, which was a disadvantage of the interpolation method. The average RMSE except for the two patches was 0.0642. The average RMSE for 24 color patches using the weighted PCA method was 0.0629. For comparison, when we applied the Wiener method to the same RGB values of the observations $y_{i}$ (*i* = 1, 2, ..., 24), the average RMSE was 0.0618. Thus, both interpolation and weighted PCA methods were inferior to the Wiener method and also to any results shown in Figure 14. The reason for this may be that the interpolation and weighted PCA methods neglected the system noise.

## 7. Conclusions

We have proposed a novel method to estimate the surface-spectral reflectance from camera responses using a local optimal reflectance dataset. The imaging system was a multispectral image acquisition system, where an RGB camera was used to capture multiple images of an object scene under multiple light sources with different illuminant spectra. The camera responses in the multispectral imaging system were described by combining the camera spectral sensitivities, surface-spectral reflectance, illuminant spectra, additive noise terms, and gain parameters.

The proposed spectral estimation method was constructed in two stages. In the first stage, the local optimal reflectance dataset was selected as a set of the most reliable candidates for the optimal estimation of the spectral reflectance from the reflectance database. We predicted the observations of the camera responses for the respective spectral reflectances in the database, which were then compared with the camera responses observed from an actual object. The optimal reflectance dataset was selected so that the prediction error was minimized. In the second stage, the best reflectance was estimated using only the local optimal dataset. We proposed multiple estimation methods of two statistical methods using the Wiener and LMMSE estimators and two mathematical programming methods using linear and quadratic programming.

Experiments were conducted using three mobile phone cameras, seven LED light sources, and a standard spectral reflectance database. First, we examined the performances of the proposed methods. All the proposed methods were significantly more accurate than the methods without using the local optimal reflectance dataset. In particular, the quadratic programming method performed much better than the other three methods. Next, we compared the proposed method with the previous methods. Our method, which involved selecting the local optimal reflectance dataset, is superior to the previous adaptive method. Additionally, we performed a comparison with the single RGB-based approach, the result of which demonstrated the superiority of the multispectral imaging approach.

We point out that the proposed approach has good features. The estimation algorithm is simple and effective. A much smaller local optimal dataset consisting of about 20 reflectances among 1776 spectral reflectance data is enough to obtain the optimal estimates. Also, the mathematical programming methods based on linear and quadratic programming without requiring noise covariance enable simpler estimations.

As the parameters of gain and noise are imaging condition dependent and multiple exposures are required, the present multispectral imaging system may not be easy to apply in open or uncontrolled illumination/lighting environments, such as the normal natural scene. Full development of an imaging system and spectral estimation method available in an open environment remains as future work.

## Data Availability

Data underlying the results presented in this paper are available at http://ohlab.kic.ac.jp/ (accessed on 10 January 2023).
